# A cross-sectoral, short-stay hospital model in general medicine (STATAMED): study protocol for a cluster-randomised, stepped-wedge controlled trial

**DOI:** 10.1186/s13063-025-09072-6

**Published:** 2025-09-16

**Authors:** Johannes Jahn, Eva-Maria Wild

**Affiliations:** 1 Hamburg Center for Health Economics, University of Hamburg, Hamburg, Germany; 2 School of Economic Disciplines, University of Siegen, Siegen, Germany

**Keywords:** General medical hospital care, Mobile nurse, Short-stay ward, New model of care, Case manager, Cross-sector care, Stepped-wedge design, Claims data, Length of stay, Readmission

## Abstract

**Background:**

Ageing populations and the increasing prevalence of chronic diseases pose major challenges to health care systems in high-income countries, especially in rural areas affected by a decline in public services. This trial investigates a new short-stay hospital model, STATAMED, which adopts a general medical approach to addressing the needs of adult patients (18 years or older) with chronic diseases and/or acute infections, with a particular focus on the elderly. STATAMED short-stay wards will incorporate a mandatory case review between the referring physician and the lead STATAMED physician, as well as support from case managers and mobile nurses to ensure coordination and continuity of care after hospital discharge. The primary aim of the trial is to determine whether the care provided in STATAMED wards is more effective than usual hospital care in improving the combined primary outcome of hospital length of stay and 30-day hospital readmission rates.

**Methods:**

The study will be conducted as a prospective, cluster-randomised controlled trial using a stepped-wedge design. STATAMED wards will be implemented at six hospital sites, and the catchment areas will be divided into clusters. All referring entities in a catchment area—including general practitioners (GPs), office-based specialists, nursing homes, ambulatory care services, and emergency services—will be potential candidates for cluster formation. The intervention group is expected to consist of 4481 patients. Primary and secondary outcomes will be assessed using claims data from participating statutory health insurers. A health economic evaluation will also be conducted using these data to compare costs associated with STATAMED and usual care. To minimise the risk of bias, analyses will be conducted on both an intention-to-treat and actual treatment basis.

**Discussion:**

This trial will assess whether STATAMED improves health care utilisation outcomes that reflect the efficiency and quality of inpatient care and discharge planning compared to usual hospital care. The findings will provide novel evidence about how best to improve health care for patients with chronic diseases and/or acute infections and may offer evidence for cost-effective strategies to improve health care delivery.

**Trial registration:**

German Clinical Trials Register DRKS00033096. Registered on 27 November 2023, last updated on 26 May 2025.

**Supplementary Information:**

The online version contains supplementary material available at 10.1186/s13063-025-09072-6.

## Administrative information

Note: The numbers in curly brackets in this protocol refer to SPIRIT checklist item numbers. The order of the items has been modified to group similar items (see http://www.equator-network.org/reporting-guidelines/spirit-2013-statement-defining-standard-protocol-items-for-clinical-trials/).

**Table:** 

Title {1}	*A cross-sectoral, short-stay hospital model in general medicine(STATAMED): Study protocol for a cluster-randomised, stepped-wedge controlled trial*
Trial registration {2a and 2b}.	German Clinical Trials Register, DRKS00033096 (WHO InternationalClinical Trials Registry Platform [ICTRP]). Registered on 27 November 2023, last updated on 15 November 2024
Protocol version {3}	Version 1 of 17 July 2025.
Funding {4}	The study is funded by the Innovation Fund of the German Federal Joint Committee, Gutenbergstraße 13, 10587 Berlin (funding no.01NVF22103). The funders have no role in the collection, analysis, or interpretation of the data, or in the preparation of manuscripts resulting from this trial.
Author details {5a}	J. Jahn: University of Hamburg, Hamburg Center for Health Economics, Esplanade 36, 20354 Hamburg; E.-M. Wild: University of Siegen, School of Economic Disciplines, Unteres Schloß 3, 57072 Siegen, Germany & University of Hamburg, Hamburg Center for Health Economics, Esplanade 36, 20354 Hamburg
Name and contact information for the trial sponsor {5b}	Prof. Dr. Eva Wild, eva.wild@uni-hamburg.de, evamaria.wild@uni-siegen.de
Role of sponsor {5c}	The funder has no role in the collection, analysis, or interpretation of the data, or in the preparation of manuscripts resulting from this trial.The study sponsor is the principal investigator, who is responsible for the design and conduct of the evaluation.

## Introduction

### Background and rationale {6a}

Ageing populations and the increasing prevalence of chronic diseases, especially in high-income countries, pose major challenges to health care systems [1, 2]. These trends not only increase demand for health care services but also require a shift in the type of care provided. Many health systems remain overly focused on acute care, which often involves surgery or advanced diagnostic technologies rather than addressing the long-term needs of chronically ill patients [3]. Another challenge exacerbating these problems is the growing difficulty of providing adequate care in rural areas. Increasing urbanisation has led to a decline in infrastructure, public services, and cultural activities in many rural regions. This trend has also affected the availability of health care services [4–6], disproportionately impacting the remaining population, especially the elderly [7, 8].

A further problem facing many health systems is the overutilisation of medical services [9–11], which “occurs when a health service is provided under circumstances in which its potential for harm exceeds the potential benefit” [12]. The potential consequences of overutilisation include rising health care costs, inefficient resource utilisation, unnecessary stress for patients, and negative health effects [11]. Overutilisation can result from various factors, including financial incentives from remuneration systems, physician uncertainty, and patient demand. A further important cause of overutilisation is a lack of coordination among different providers of care [13, 14], particularly in countries with fragmented health care systems, such as Germany [15], where this trial is taking place. The elderly and chronically ill are especially vulnerable to the effects of fragmented care because their treatment generally requires a higher degree of coordination [16, 17].

The current trial evaluates a new model of care, referred to as STATAMED, which aims to address the challenges outlined above. At participating hospitals, a general medicine short-stay ward (“STATAMED ward”) with approximately 20 beds will be integrated into the existing hospital infrastructure. Two implementation models can be distinguished: (1) Hospitals in which a STATAMED ward is established and operates alongside existing wards and (2) hospitals in which the STATAMED ward constitutes the sole inpatient ward. In addition to standard general medical treatment, STATAMED wards will include interdisciplinary treatment planning before hospital admission (facilitated through a mandatory case review between the referring physician and the lead STATAMED physician), as well as support from case managers and mobile nurses during and after the hospital stay to ensure coordination and continuity of care after hospital discharge.

The mandatory case review between the referring outpatient physician and the lead STATAMED physician is conducted to determine patient eligibility for the trial and to collaboratively plan the treatment. While the literature supports the benefits of better-integrated models of service delivery [18], mandatory case reviews between referring physicians and lead physicians at receiving hospitals are currently neither widely implemented nor thoroughly studied.

Short-stay units are employed in various medical contexts, including paediatric care, surgical care, emergency care, and general medicine. Depending on the setting, the intended length of stay in short-stay units is typically 5 days or fewer [19]. In a meta-analysis, Damiani et al. [19] found that short-stay units reduced length of stay without adversely affecting mortality and readmission rates. However, a literature review by Strøm et al. [20] focusing on general medical conditions concluded that current evidence regarding reduced mortality, serious adverse events, and rehospitalisation is insufficient to definitively assess the benefits and harms of short-stay units.

The primary motivation for deploying case managers is to improve care coordination, which involves communication with various health care providers, social service providers, and the patients’ relatives. A literature review by Joo et al. [21] found that case managers reduce readmissions, hospital visits, emergency department visits, and length of stay.

Mobile nurses are introduced to ensure proper follow-up care after hospital discharge. Kwok et al. [22] analysed the effectiveness of mobile nurses for older patients with chronic heart failure, reporting positive effects on patient independence and indications of reduced readmissions, although no evidence of cost-effectiveness was found. Chow et al. [23] examined mobile nurse services covering various chronic diseases and identified positive effects on self-reported health status but no impact on readmission rates.

Conducting this trial is important to assess whether STATAMED contributes to improvements in care. Evaluating innovative care models is critical for determining which new approaches should be adopted as part of routine care. This is particularly important given the budget constraints that most health care systems face and their obligation to allocate public funds effectively and efficiently. This consideration is especially relevant to the statutory health insurance system in Germany, where health expenditures per capita are among the highest in Europe, yet health outcomes are not always commensurate with this level of spending [24]. This trial is also highly relevant in light of the current challenges facing the German hospital sector. The hospital market in Germany is characterised by excess capacity in terms of hospital beds [25] and a concurrent shortage of nursing staff [26, 27]. A reform of the hospital sector is currently being planned, with the dual goal of reducing the number of hospital sites and improving the quality of care in the remaining facilities. One focus of this reform concerns the role of small hospitals, particularly those in rural areas. Proponents argue that these hospitals are vital for ensuring timely access to inpatient care, whereas critics contend that they struggle to maintain sufficient quality due to low patient volumes. In this context, STATAMED may provide a viable solution for maintaining broad access to general medical care in hospital settings while addressing inefficiencies and improving health outcomes.

## Objectives {7}

The primary aim of this study is to investigate whether STATAMED reduces the length of hospital stay and 30-day hospital readmission rates (combined primary outcome) in the target population, which comprises adult patients with chronic diseases and/or acute infections (see the “Eligibility criteria {10}”). To achieve this, the following interventions will be implemented: a mandatory case review between the referring physician and the lead STATAMED physician to determine patients’ eligibility for the trial and to collaboratively plan their treatment, standard general medical care delivered in a general medical short-stay hospital ward with approximately 20 beds, discharge planning and follow-up care coordinated by case managers, and post-discharge care delivered by mobile nurses (see the “Intervention description {11a}” for details).

During the case review, diagnoses will be reviewed, and a treatment plan will be agreed upon with the goal of minimising unnecessary diagnostic procedures, avoiding overdiagnosis, and ultimately shortening the hospital stay. Reducing the length of stay is expected to lower the risk of nosocomial complications such as hospital-acquired infections, delirium, immobility, and thromboses, all of which can prolong hospitalisation [28–30].

The 30-day readmission rate will be assessed as part of the primary outcome because it is a widely used and reliable indicator of care quality [31]. The assumption is that if reducing the length of stay through STATAMED compromises the quality of care, this would be reflected in higher readmission rates. However, Damiani et al. [19] found that short-stay wards can reduce length of stay without adversely affecting mortality or readmission rates. Furthermore, follow-up care provided by STATAMED, including support from case managers and mobile nurses, is also expected to reduce readmission rates [21]. By combining these two outcomes—length of hospital stay and 30-day hospital readmission rate—this study aims to evaluate whether a reduction in hospital stay can be achieved without compromising the quality of care for the target population.

In addition, several secondary outcomes will be analysed: utilisation of hospital emergency department, emergency outpatient care, and non-emergency outpatient care, as well as the place of discharge. These outcomes were chosen to gain a more comprehensive understanding of the entire patient pathway in relation to the treatment provided at each STATAMED ward. They are also relevant for assessing the cost-effectiveness of STATAMED.

We propose three additional hypotheses regarding the impact of STATAMED on secondary outcomes:

1. Reduction in hospital emergency admissions and utilisation of emergency outpatient care: We hypothesise that STATAMED will reduce hospital emergency admissions and the utilisation of emergency outpatient care after initial discharge through improved coordination and the provision of follow-up care. This hypothesis is supported by findings from a systematic review by Raven et al. [32], which suggest that case management interventions can reduce emergency department visits.
2. Non-emergency outpatient care utilisation: We expect two opposing effects on non-emergency outpatient care utilisation. Follow-up care provided by the mobile nurse may substitute for certain outpatient services, whereas the coordination of follow-up care by the case manager, including scheduling appointments with outpatient physicians, is expected to increase the utilisation of non-emergency outpatient care.
3. Increase in discharges to home rather than nursing care facilities: We also hypothesise that patients treated at STATAMED wards will be more likely to be discharged to their homes rather than to nursing care facilities. This is attributed to improved treatment quality, which is expected to prevent overtreatment and reduce the risk of nosocomial and other complications.

A health economic evaluation will be conducted to assess the cost-effectiveness of STATAMED. We hypothesise that the enhanced hospital care provided in STATAMED wards will be more cost-effective than usual hospital care. Reducing the length of hospital stay is expected to lower costs for health insurers. Readmission rates also serve as an indicator of cost-effectiveness [31]. For example, Jencks et al. [33] estimated that unplanned readmissions of Medicare beneficiaries in 2004 cost US $17.4 billion. Reduced utilisation of emergency outpatient care is also associated with lower health care costs. Whereas increased utilisation of outpatient care may result in higher costs for outpatient treatments, it can help prevent costly readmissions to hospital.

## Trial design {8}

The STATAMED study will be conducted as a prospective, cluster-randomised controlled trial (C-RCT) using a stepped-wedge design at six hospital sites. The catchment areas of the participating sites will be divided into clusters based on postal codes. Clusters were formed using the number of referring entities (e.g. GPs, specialists, nursing homes, ambulatory care services, and emergency services) as the criterion, with the goal of creating a sufficient number of clusters of approximately equal size to facilitate analysis and ensure sufficient statistical power [34].

**Fig. 1:**
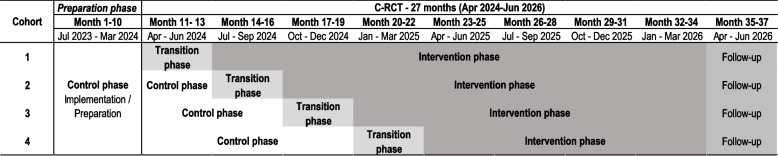
Study design

The stepped-wedge design entails each cluster beginning in a control phase and sequentially transitioning to the intervention phase in staggered steps. Each cluster undergoes a 3-month transition phase to practice and refine the intervention before fully entering the intervention phase. The shift from the control phase to the transition phase is divided into four steps, with one-quarter of the clusters transitioning every 3 months. This staggered approach allows intervention effects to be recorded and compared over time [35]. Once a cluster transitions into the intervention phase, it will remain in this phase for the duration of the study. After the intervention phase, a follow-up phase will be conducted to complete all outcome measurements. Figure 1 illustrates the study design.

The intervention group consists of all eligible patients (see the “Eligibility criteria {10}”) treated in the STATAMED wards. Eligible individuals must be covered by one of the participating statutory health insurers and meet the eligibility criteria. Claims data will only be provided for individuals insured by one of the participating statutory health insurers.

**Fig. 2:**
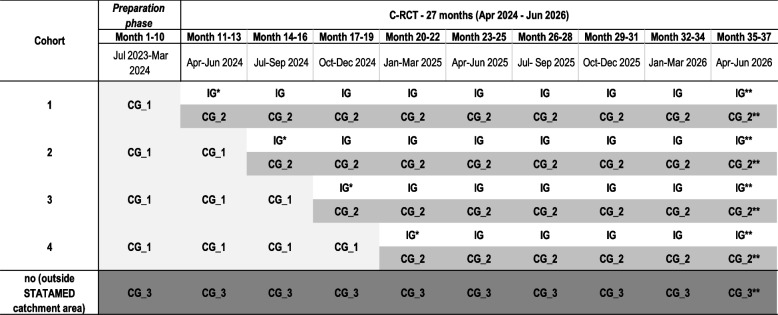
Intervention groups and control groups. Notes: IG—intervention group, CG_1—control group 1, CG_2—control group 2, CG_3—control group 3, *transition phase,** follow-up

Additionally, three control groups are planned:

- Control group 1 consists of patients who meet the eligibility criteria for treatment in a STATAMED ward but receive usual hospital care at a non-STATAMED hospital because their referring entity is located in a cluster assigned to the control phase at the time of treatment. This is the standard control group in the stepped-wedge design, enabling the analysis of each cluster before and after STATAMED is introduced.
- Control group 2 consists of patients who meet the eligibility criteria for treatment at a STATAMED ward and whose referring entity is located in a cluster assigned to the intervention phase at the time of treatment but who are referred to and receive treatment at a non-STATAMED hospital. This group allows for the examination of possible selection effects (i.e. patients who prefer usual hospital care over STATAMED).
- Control group 3 consists of patients who meet the eligibility criteria for treatment at a STATAMED ward but whose referring entity is located outside a participating hospital site’s catchment area, resulting in treatment at a non-STATAMED hospital. This group allows for the consideration of additional spatial effects. Figure 2 illustrates the various control groups.

## Methods: participants, interventions, and outcomes

### Study setting {9}

STATAMED will be implemented at six hospital sites across Germany, with three located in rural areas and three in urban areas. The list of study sites can be found in the registry (DRKS00033096).

The six sites consist of small- and medium-sized hospitals, with the smallest having 15 beds and the largest 229 beds. The sites also vary in other characteristics, such as the number of wards present in addition to the STATAMED ward and whether the hospital has an emergency department. This information is available to the evaluators and will be taken into account during the analysis.

Data for the control group will be obtained from patients who receive treatment at non-STATAMED hospitals. These may be any hospital that is allowed to treat patients who are covered by statutory health insurance (see also “Explanation for the choice of comparators {6b}”).

### Eligibility criteria {10}

#### Inclusion criteria for patients

Patients are eligible for inclusion if they meet the following criteria: They present at a participating referring entity with a general medical condition requiring inpatient treatment as defined by the German Appropriate Evaluation Protocol (G-AEP), have a diagnosis corresponding to an ICD-10 code designated specifically for this project (i.e. conditions relevant to the trial), and are expected to have an uncomplicated course of disease corresponding to a patient clinical complexity level (PCCL) of 0 to 2. Additionally, patients must be at least 18 years of age.

The following list of ICD-10-GM (2023) codes includes exemplary diagnoses relevant to the study but is not exhaustive:

- I10.01 (benign essential hypertension: with mention of hypertensive urgency)
- I48.1 (persistent atrial fibrillation)
- E86 (volume depletion)
- K59.09 (other and unspecified constipation)
- I50.14 (left ventricular failure: with symptoms at rest)
- I20.8 (other forms of angina pectoris)
- I48.0 (paroxysmal atrial fibrillation) and R55 (syncope and collapse)
- R07.3 (other chest pain) and K29.6 (other gastritis)
- N39.0 (urinary tract infection, site not specified)
- I50.13 (left ventricular failure: with symptoms under mild strain)
- J20.9 (acute bronchitis, unspecified)
- J18.9 (pneumonia, unspecified)
- I50.01 (secondary congestive heart failure)
- K57.32 (diverticulitis of large intestine without perforation, abscess, or bleeding)
- E11.91 (type 2 diabetes mellitus: without complications, uncontrolled)
- R10.3 (pain localised to other parts of lower abdomen)
- A09.9 (gastroenteritis and colitis of unspecified origin)
- K29.1 (other acute gastritis)

The complete list of ICD-10 codes can be provided upon request.

#### Exclusion criteria for patients

Patients will be excluded if they meet any of the following criteria: They require highly technical diagnostic procedures (as defined by the eligibility criteria for STATAMED wards), require inpatient surgery, or have been transferred from other hospitals.

#### Eligibility criteria for STATAMED wards

Each participating hospital site is required to ensure the availability of a defined minimum number of staff for its STATAMED ward, including 2.9 full-time equivalent (FTE) ward physicians along with frontline on-call coverage and backup coverage by a senior physician, as well as 7.5 FTE nurses. The following essential medical equipment must also be available: ambulatory ECG (e.g. Holter monitor), ambulatory blood pressure monitor, mobile X-ray unit, ultrasound unit, blood gas analyser, gastroscope, colonoscope, and basic laboratory facilities (cooperation with external providers is permissible). These staffing and equipment requirements are based on a STATAMED ward capacity of 20 beds.

Participation in the project is voluntary. However, hospitals that agree to participate and implement a STATAMED ward are contractually obliged to meet the eligibility criteria throughout the trial period. If a participating a hospital voluntarily withdraws from the project or is no longer able to meet the criteria (e.g. due to staff shortages), the funder may require repayment of the financial support provided for STATAMED personnel. This includes staff funding for the lead STATAMED physician, case manager, and mobile nurse.

Any such change in participation status must be reported immediately to the health insurer leading the project consortium and to the evaluators, who will assess the implications for ongoing trial operations and determine whether corrective measures are required (e.g. recruitment adjustments). The evaluation team will document and account for these events during the final analysis. Patient safety is ensured as STATAMED services are offered in addition to standard care.

If a STATAMED ward is no longer able to provide standard care services, all patients currently receiving treatment will be transferred to an appropriate ward or facility to ensure that their care is not disrupted.

#### Eligibility criteria for referring entities

Referring entities, including GPs, office-based specialists, nursing homes, ambulatory care services, and emergency services, must complete mandatory training on STATAMED-specific processes, such as the case review process, prior to participation. Additionally, they must sign a participation agreement to be authorised to refer patients to STATAMED wards.

Participation is voluntary and may be discontinued at any time. Referring entities that choose to withdraw from the project may cease referring patients to STATAMED wards without further consequences. They may also revoke consent for the use of their practice-related data in the scientific evaluation, in accordance with applicable data protection regulations. In the event of withdrawal, the health insurer leading the project consortium should be notified to ensure accurate documentation and appropriate adjustments to recruitment planning.

### Who will take informed consent? {26a}

Informed consent from trial participants in the intervention group will be obtained during the admission process at the STATAMED wards.

The three control groups consist of patients who meet the eligibility criteria but receive treatment at non-STATAMED hospitals. The control groups will be identified using claims data from the participating statutory health insurers. The evaluation of outcomes in the control groups will be based on routinely collected claims data, which the participating statutory health insurers provide independently of the study. The claims data will be anonymised, and no informed consent is required for these groups.

### Additional consent provisions for collection and use of participant data and biological specimens {26b}

No personal data are collected. The claims data used for this study are anonymised. No biological specimens will be collected in this trial.

### Interventions

#### Explanation for the choice of comparators {6b}

STATAMED is a new model of care that provides standard general medical treatment in a short-stay ward with approximately 20 beds, combined with various other care coordination interventions (see below). To assess its effectiveness, the hospital care provided in STATAMED wards will be compared to usual hospital care (i.e. standard general medical treatment only).

In Germany, hospital treatment is regulated by Book V of the German Social Code (SGB V). Hospital admission is restricted to cases that cannot be adequately managed by outpatient physicians. Nonemergency patients must be referred for admission by an outpatient physician. Patients insured through statutory health insurance, which covers 87.3% of the German population [36], may choose any hospital as long as the hospital is part of the state hospital plan (“Landeskrankenhausplan”), a university hospital, or has a contract with statutory health insurers (“Versorgungsvertrag”). These conditions are met by 89% of hospitals in Germany [37].

### Intervention description {11a}

STATAMED encompasses a range of interventions designed to ensure continuity of care and improve coordination between hospital and outpatient settings both before and after hospital discharge. The following interventions will be implemented:

1. Short-stay ward: Each participating hospital site will include (or, in the case of very small hospitals, consist of) a dedicated short-stay ward (“STATAMED ward”) for patients with general medical conditions who require inpatient treatment, have a diagnosis matching a STATAMED-specific ICD-10 code, and are expected to have an uncomplicated disease course (see eligibility criteria {10}). The average length of stay in a STATAMED ward is anticipated to be approximately 3 days.
2. Case review: To determine whether a patient is suitable for treatment in a STATAMED ward, a mandatory case review will be conducted between the referring physician and the lead STATAMED physician of the respective STATAMED ward. During this review, diagnoses will be assessed and discussed, eligibility will be confirmed, and a treatment plan will be collaboratively developed. If the lead STATAMED physician approves the admission, a planned admission will be scheduled, specifying the date and time to ensure that the STATAMED ward can prepare for the patient’s arrival and admit him or her without delay.
3. Case manager: Case managers in each STATAMED ward will coordinate interdisciplinary and cross-sectoral treatment planning for the post-discharge period. During the hospital stay, they will participate in medical rounds to support discharge planning. Their responsibilities include assisting patients with urgent social care needs, such as arranging home care services, procuring assistive devices, and securing short-term care home placements. After discharge, case managers will help coordinate necessary therapeutic measures, including organising nursing services, scheduling physiotherapy sessions, arranging home visits by occupational therapists, and making appointments with outpatient physicians. They will also communicate with and involve the patient’s caregivers and relatives as needed. Case managers hold a basic qualification in a medical, therapeutic, nursing, or social care profession (e.g. medical assistants) and have received project-specific training to prepare them for their tasks in the STATAMED trial.
4. Mobile nurses: These nurses will provide follow-up care either at the patient’s home or in a long-term care facility. Their responsibilities include medical tasks such as monitoring vital signs, collecting blood samples for analysis, and performing ultrasounds or electrocardiograms. They also assess patients for functional deficits, provide fall prevention guidance, and report on follow-up care needs. Additionally, mobile nurses can assist with the admission process. If a patient’s regular referring physician is unavailable, the mobile nurse can visit the patient before admission to assess their condition. In such cases, the lead STATAMED physician will be consulted via telemedicine to make the admission decision, and the mandatory referral case review will take place at a later date.

All patients who receive treatment in a STATAMED ward will receive the full set of trial interventions without further differentiation within the intervention group. However, certain services offered by the case manager or mobile nurse (e.g. follow-up care) may not be medically necessary for some patients, may be declined by some patients, or may not be provided due to capacity constraints. All services provided will be documented, and STATAMED staff will also document any requested services that could not be delivered (e.g. due to capacity constraints).

### Criteria for discontinuing or modifying allocated interventions {11b}

Patients will be transferred from a STATAMED ward to a non-STATAMED hospital if their medical condition worsens or if new symptoms arise that require additional medical equipment, critical care, surgery, specialised medical care, or further diagnostic procedures beyond the capabilities of the STATAMED ward.

### Strategies to improve adherence to interventions {11c}

The case review between the referring physician and lead STATAMED physician is a central component of the trial intervention and is mandatory. However, in certain situations, scheduling the review before admission may not be feasible (e.g. when patients are transferred from other wards within the same hospital to the STATAMED ward outside the working hours of the lead STATAMED physician). In such cases, the review will be conducted as soon as possible after admission. If it is determined that a patient does not meet the eligibility criteria, he or she will be transferred to another ward or hospital. The completion and details of the review must be documented.

The case manager and the mobile nurse are required to document their activities.

### Relevant concomitant care permitted or prohibited during the trial {11d}

This is not applicable. After being admitted to a STATAMED ward, patients receive standard general medical treatment according to their medical needs. No specific drug or medical procedure is part of the intervention.

### Provisions for posttrial care {30}

This is not applicable. Beyond the care coordination components of the STATAMED intervention, patients in the intervention group also receive standard general medical treatment. No risks are anticipated with treatment at STATAMED because the mandatory case review ensures that only patients who can be adequately treated in a STATAMED ward are admitted.

### Outcomes {12}

All outcomes will be measured using claims data from the participating statutory health insurers. A detailed description of the claims data is provided below (see “Plans for assessment and collection of outcomes {18a}”).

The combined primary outcome consists of the following:

(1) Length of hospital stay, which is measured in days and calculated as the difference between the admission date and discharge date. (2) A 30-day hospital readmission rate, which captures the number and length of hospital stays within 30 days after discharge

The secondary outcomes are as follows:

- Utilisation of hospital emergency departments
- Utilisation of emergency outpatient care
- Utilisation of nonemergency outpatient care
- Place of discharge (discharge to patient home vs. discharge to a care facility)

Utilisation of hospital emergency departments, utilisation of emergency outpatient care, and utilisation of nonemergency outpatient care will be measured in monetary terms and by the number of services provided. These outcomes will be measured before and after the hospital stay.

A health economic evaluation will also be conducted to assess the cost-effectiveness of STATAMED.

### Participant timeline {13}

The participant timeline consists of three phases:

- Pre-hospital phase: The timeline starts before the hospital stay with an interaction between a potential participant and a referring entity (e.g. a GP), during which the need for hospitalisation is assessed. If the referring entity determines that the potential participant meets the eligibility criteria for the intervention, the STATAMED ward is contacted for the mandatory case review between the referring physician and the lead STATAMED physician. If the eligibility criteria are met, the admission is scheduled to take place approximately 1–2 days later.
- Hospital phase: Upon provision of informed consent and admission to the STATAMED ward, participants are formally enrolled in the study. The hospital stay is expected to last approximately 3 days on average. During this period, patients receive standard general medical treatment in the STATAMED unit, and case managers assist with interdisciplinary care planning and discharge preparations.
- Posthospital phase (up to 28 days after discharge): After discharge, follow-up care is available. The mobile nurse may visit the patient at home or in a care facility. Case managers can assist patients during the hospital stay and after discharge, coordinating follow-up care and addressing post-discharge needs.

**Fig. 3:**
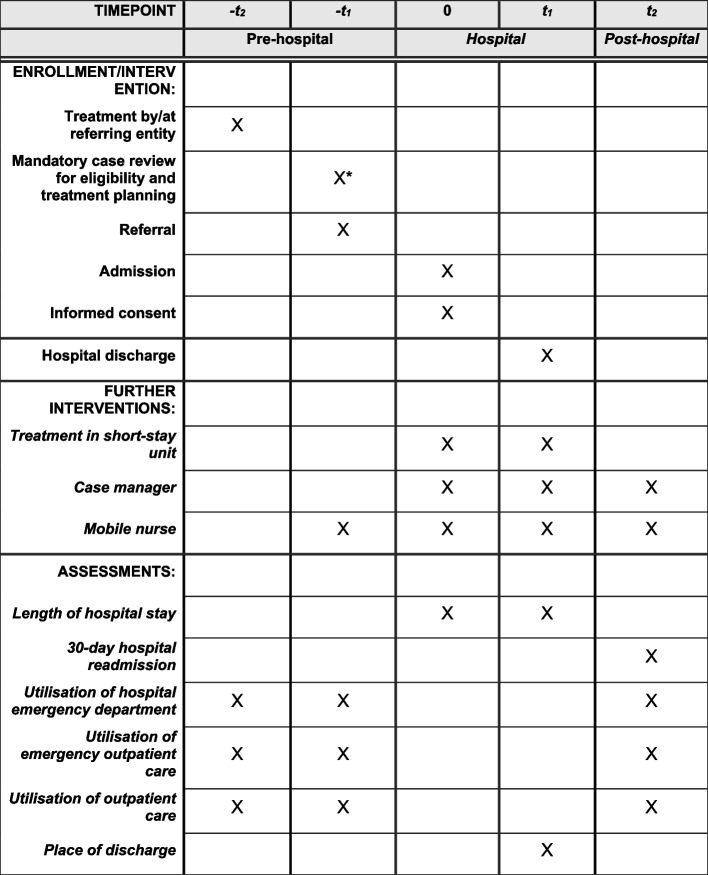
Participant timeline. Notes: *The case review takes place between the referring entity and the lead STATAMED physician

Figure 3 illustrates the participant timeline, covering the pre-hospital, hospital, and posthospital phases.

All assessments of primary and secondary outcomes will be conducted using claims data from statutory health insurers. Data collection does not require any direct involvement from participants.

### Sample size {14}

The required sample size was estimated prior to project implementation using Bonferroni correction to account for multiple comparisons because the trial includes two co-primary outcomes without a hierarchical structure: (1) length of hospital stay and (2) 30-day hospital readmission. Applying a type 1 error rate of *α* = 5%, the Bonferroni correction results in a per-outcome significance level of α/2 = 2.5% for confirmatory testing.

Sample size calculations were performed according to Woertman et al. [38]. Based on existing literature [39] and a pre-analysis of claims data, a minimum relevant difference of 15% was specified for both co-primary outcomes. An intra-class correlation coefficient (ICC) of *ρ* = 0.05 was assumed to account for within-cluster correlation [40, 41], resulting in a design effect estimate of 0.61.

For co-primary outcome (1), the required sample size was estimated at 2331 participants; this increases to 2681 participants after adjusting for a 20% drop-out rate. For co-primary outcome (2), the required sample size was estimated at 3724 participants, rising to 4481 after applying the same drop-out assumption. Given that both outcomes are co-primary, the larger of the two estimates (i.e. 4481 participants) was initially adopted as the required sample size for the intervention group across all clusters over the full duration of the study.

These initial calculations relied on conservative assumptions, including a high estimated drop-out rate and the use of the Bonferroni correction for multiple comparisons. However, evidence from the literature and ongoing implementation experience allow for the following refinement of these assumptions.

Based on recent experience with implementation logistics and recruitment patterns, a more realistic drop-out rate of 15% is now assumed. In addition, Leppin et al. [42] conducted a meta-analysis that found an 18% reduction in the risk of readmission. Complex interventions and interventions ensuring structured follow-up care are found to be particularly effective in reducing readmission. Because both elements are core features of STATAMED, an effect size of 20% is considered plausible.

Finally, to adjust for multiple comparisons, the Romano-Wolf method [43] offers an alternative to the Bonferroni correction. This method controls the family-wise error rate using a step-down bootstrap procedure that accounts for the correlation among hypotheses, thereby making more efficient use of the global *α* level. Applying this method permits the use of a global significance level of 5% for sample size calculation.

Under these updated assumptions (i.e. 20% effect size, 15% drop-out rate and Romano-Wolf correction), the required sample size is reduced to 2076 participants for the readmission outcomes and 578 for length of stay. Accordingly, 2076 participants are required for evaluating the combined primary outcome.

The sample size calculation refers to the required number of participants for whom claims data are available. This is important because claims data are only available for patients insured by one of the participating statutory health insurers.

### Recruitment {15}

Participant recruitment will be carried out by the referring entities in the catchment area of each participating hospital site. To achieve the target sample size, a sufficient number of referring entities must be involved. Therefore, the lead STATAMED physician at each intervention site is responsible for establishing a local network of referring entities to support recruitment. Lead STATAMED physicians will contact potential referring entities individually and inform them about the project. In addition, information events will be held, and public outreach (e.g. press releases, radio and TV interviews, and social media campaigns) will be used to raise general awareness of the project.

Referring entities must sign a contract to participate in STATAMED and obtain authorisation to refer patients. There is no minimum number of referring entities required per hospital as local conditions (e.g. population size and physician density) vary across sites. Each hospital is expected to recruit a sufficient number of referring entities to ensure that the required sample size is reached.

### Assignment of interventions: allocation

#### Sequence generation {16a}

The assignment of clusters to the time point when the transition phase starts will be randomised. However, an exception to randomisation is necessary at the beginning of the study. At the participating hospital sites, staff must be hired, and facilities need to be prepared before the intervention can begin. Thus, it must be ensured that at least one cluster at each participating hospital site is assigned to the intervention group at the beginning of the study. The selection of the first cluster at each site will be randomised.

The assignment of the remaining clusters in subsequent steps will follow a fully randomised approach, with the restriction that a maximum of one cluster per STATAMED ward will be assigned at each step. This restriction ensures that the number of participants per STATAMED ward develops evenly over the course of the trial.

These exceptions to randomisation are necessary to ensure the feasibility and acceptance of the research design at the STATAMED wards.

Randomisation will be based on computer-generated random numbers.

### Concealment mechanism {16b}

This is not applicable. Patients who meet the eligibility criteria and whose referring entity is located in a cluster assigned to the transition or intervention phase can participate in STATAMED. Before admission to a STATAMED ward, patients must provide informed consent to participate in the project.

The control groups will consist of patients who meet the eligibility criteria but receive treatment at a non-STATAMENT hospital. They will be identified retrospectively based on ICD-10 codes available in the claims data from the participating health insurers.

### Implementation {16c}

The allocation sequence will be generated through randomisation (see “Sequence generation {16a}”). The randomisation process will be conducted by the authors.

Participants will be enrolled at their respective STATAMED wards. Assignment to the intervention will depend on the cluster to which the referring entity is assigned. Patients who meet the eligibility criteria and whose referring entity is in a cluster currently in the intervention phase will be able to participate in STATAMED.

### Assignment of interventions: blinding

#### Who will be blinded? {17a}

This is not applicable. Blinding is neither possible nor required for the interventions evaluated in this trial.

### Procedure for unblinding if needed {17b}

This is not applicable. No procedure for unblinding is required, as neither participants nor STATAMED staff are blinded.

### Data collection and management

#### Plans for assessment and collection of outcome {18a}

Claims data from the statutory health insurers participating in the trial (AOK Rheinland-Hamburg and AOK Niedersachsen) will be used as the primary data source. Claims data are secondary data collected by health insurers for billing purposes and are considered reliable and valid. Because they reflect actual treatments provided by physicians and hospitals, they contribute to ensuring external validity. Matching methods will be applied in the analysis to improve comparability between the intervention and control groups, minimising selection bias [44].

To ensure data quality, data will be delivered at two time points by the participating health insurers. The final data delivery is expected at the end of the project period (approximately January 2027). Before this, a preliminary data delivery will take place (approximately June 2026) to check the completeness and plausibility of the data.

Although the outcomes will be measured using claims data, additional data will be collected to ensure the feasibility of the analysis. Table 1 provides an overview of the various data sources.

**Table 1:** Data sources

Data set	Description	Utilisation
Claims data from participating statutory health insurers	Secondary data collected by statutory health insurers for billing purposes. The data set includes: patient master data (e.g., sex, year of birth, postal code), outpatient data (e.g., pseudonymised physician number, diagnoses[ICD codes], procedures, billed services), medicines (e.g., ATC code, issuance date, price/amount of reimbursement), inpatient data(e.g., hospital number, diagnoses [ICD-code], procedures, date of inpatient admission and duration of hospital stay, billed case rates)	Measuring primary andsecondary outcomes
Task documentation data	STATAMED-specific tasks performed by the lead STATAMED physician, mobile nurse, and case manager must be documented (e.g., mobile nurses document tasks performed during follow-up care, such as measuring vitalsigns or taking blood samples)	Allows assessment and comparison of implementationacross different STATAMED wards
Information on referring entity	Identification numbers and postal codes of referring entities	Allows assignment of patientlevel claims data to the correct cluster
Data according to §21 KHEntgG	Data provided by hospitals with STATAMED wards, including structural data (e.g., type of hospital, number of beds, number of nursing staff, number of physicians) and performance data (e.g., patient information [age, gender,residence], admission date, discharge date, diagnoses [ICD codes], procedures, billed services)	Measuring length of hospital stay for participants not enrolled with aparticipating health insurer

### Plans to promote participant retention and complete follow-up {18b}

Analyses will be based on claims data that are automatically collected when patients use medical services that are covered by the participating statutory health insurers. For this reason, no measures to ensure follow-up are required.

Patients are free at any time to withdraw their consent to participate in the study. In such cases, their data will be excluded from analysis.

STATAMED wards are equipped and staffed to treat patients who meet the eligibility criteria with appropriate, high-quality care. However, if a patient’s medical condition worsens or additional diagnostics are required during treatment, he or she will be transferred to another hospital that can provide the appropriate care. In such cases, data can still be used for analysis unless participants explicitly withdraw their consent.

### Data management {19}

Data will be transferred in encrypted form from the health insurers to the evaluators. Only authorised project members will have access to the data. Data will be stored on a secure research server, with backups made on a regular basis. All steps in data preparation and analysis will be fully documented. Raw data will be stored separately from the processed study data to ensure the replicability and integrity of the results. Data will be archived for 10 years after the study has ended.

### Confidentiality {27}

Data will be provided in anonymised form, and it will not be possible for the researchers to identify individual patients. As a result, no patient-identifying information will be reported in any publications.

### Plans for collection, laboratory evaluation, and storage of biological specimens for genetic or molecular analysis in this trial/future use {33}

This is not applicable. No biological specimens will be collected in this trial.

### Statistical methods

#### Statistical methods for primary and secondary outcomes {20a}

The two co-primary outcomes (length of hospital stay, 30-day hospital readmission rate) are treated as continuous variables. The secondary outcomes (utilisation of hospital emergency departments, emergency outpatient care, and nonemergency outpatient care, as well as place of discharge) are also continuous except for the place of discharge (home, care facility, other), which is categorial.

Because there are two co-primary outcomes without hierarchical ordering, a correction for multiple testing is required. A Bonferroni correction will be applied, resulting in a one-sided significance level of α/2 = 2.5% for each primary outcome given a global type I error rate of *α* = 5%. However, the Bonferroni method is known to be conservative. As an alternative, the Romano-Wolf procedure [43] may be used, as it more efficiently controls the family-wise error rate by accounting for the dependence structure among outcomes through resampling (see “Sample size {14}”).

When analysing stepped-wedge designs, it is important to account for temporal effects given that each cluster transitions to the intervention in a stepwise fashion. In this study, this is particularly important because the STATAMED wards will gain experience with implementing the intervention over time. Additionally, the longitudinal design of the study necessitates the consideration of calendar time because patient case mix may vary by season (e.g. winter versus summer) due to the focus of the study on general medical conditions. To account for these temporal effects, generalised linear mixed models (GLMMs) and generalised estimating equations (GEEs) will be applied [45–47]. Propensity score matching, considering age, gender, and morbidity, will be used to ensure comparability between the control and intervention groups.

To avoid bias from patients who are transferred to other hospitals during STATAMED treatment or those who meet eligibility criteria but prefer to be treated at a non-STATAMED hospital, the analysis will be conducted according to the intention-to-treat and per-protocol principles [48].

### Interim analyses {21b}

No interim analyses are planned.

### Methods for additional analyses (e.g. subgroup analyses) {20b}

To investigate the heterogeneity of treatment effects, various subgroup analyses are planned. These analyses will examine sociodemographic characteristics (age, gender, place of residence) and adherence to the intervention.

### Methods in analysis to handle protocol nonadherence and any statistical methods to handle missing data {20c}

At the patient level, nonadherence is expected to be minimal because no specific procedures are strictly required of patients once they have decided to participate in STATAMED. Furthermore, claims data are collected by the participating statutory health insurers independently of the progress of the study, making data delivery and quality independent of patient behaviour.

However, nonadherence may occur due to differences in how the various intervention components are implemented in the STATAMED wards. To address this, STATAMED staff members are required to document their tasks, including evidence of the mandatory case review between the referring entity and the lead STATAMED physician before the admission. The mobile nurse and case manager are also required to document their activities. Likewise, STATAMED wards document any request for components of the intervention that could not be carried out (e.g. due to capacity constraints). This information makes it possible to identify and evaluate nonadherence caused by implementation differences at the STATAMED wards.

Nonadherence can also occur at the level of the referring entity, particularly if patients are referred who do not meet the eligibility criteria. Although a mandatory case review is conducted before admission, the lead STATAMED physician cannot verify the accuracy of the information provided by the referring entity until the patient is admitted. If patients are wrongly admitted due to incorrect information provided by the referring entity, these patients will be transferred to a non-STATAMED hospital.

A fundamental challenge in preparing the data is missing information. Because administrative billing data are collected from a wide range of stakeholders (e.g. doctors, pharmacists, hospitals, medical supply stores), inconsistencies and missing data inevitably occur—both from errors early in the documentation process (e.g. incorrectly issued prescriptions) and from problems in later phases of data collection. When handling missing data, each case is reviewed to determine whether imputation is appropriate and, if so, which method should be employed.

As mentioned above (see “Plans for assessment and collection of outcome {18a}”) data will be delivered by the participating health insurers at two time points. The preliminary data delivery allows for checks on completeness and plausibility. This process helps minimise the amount of missing data.

### Plans to give access to the full protocol, participant level-data, and statistical code {31c}

Participant-level data are subject to data protection regulations and cannot be made publicly available. Most of the data belong to the statutory health insurers participating in the project. To access these data, researchers must obtain permission for a specific research question from the German Federal (Social) Insurance Office. Additionally, researchers must enter into a contractual agreement with the statutory health insurers regarding data access.

Statistical code can be provided upon request.

### Oversight and monitoring

#### Composition of the coordinating centre and trial steering committee {5d}

The trial will be implemented at six hospital sites across Germany. At each site, a designated physician will be responsible for the informed consent and enrolment of participants in the STATAMED ward. Coordination across the sites will be facilitated through regular meetings overseen by the health insurer leading the project consortium. These meetings will ensure that the intervention is implemented consistently at all sites.

The evaluation of STATAMED encompasses several sub-studies, with multiple research institutes involved in the project. The present protocol covers only one of these sub-studies. This protocol describes the analysis of the two co-primary outcomes measured in this trial (length of stay and 30-day readmission) using claims data. Additional components of the overall evaluation include patient-reported outcomes (e.g. patient experiences, health-related quality of life) [49] and qualitative studies on implementation processes [50], which will be reported separately.

The participating research institutes hold regular meetings to plan, prepare, and coordinate the various data collection activities. The participating health insurers also take part in these meetings.

The responsibility for evaluating the STATAMED project lies solely with the researchers. The participating hospitals and health insurers have no role in the evaluation of the invention, including the methodology used, the collection, management, analysis or interpretation of the data, in the writing or review of any related manuscripts, or in decisions regarding the publication of the results.

### Composition of the data monitoring committee and its role and reporting structure {21a}

No data monitoring committee is needed. The researchers are not blinded, and all data will be received in anonymised form.

### Adverse event reporting and harms {22}

No specific risks are associated with participation in the project. However, patients’ medical conditions may worsen, and the diagnostic equipment available in a STATAMED ward may be insufficient in some cases. In such situations, patients will be transferred to other facilities, and both the transfer and its reasons will be documented.

### Frequency and plans for auditing trial conduct {23}

Not applicable. No independent auditing will be conducted. Core trial processes will be reviewed by the participating statutory health insurers. In addition, the institutions responsible for the accompanying scientific research have conducted site visits and training sessions to ensure that the intervention is implemented as planned.

### Plans for communicating important protocol amendments to relevant parties (e.g. trial participants and ethical committees) {25}

If protocol amendments are necessary, the responsible ethics committee will be informed, the trial registration will be updated, and an updated version of the study protocol will be published.

### Dissemination plans {31a}

At the end of the project, an evaluation report will be published on the website of the funder. The report will be written by the evaluators, and the funder will not have any influence on the content of the report.

Additionally, the results will be published in international peer-reviewed journals. Positive and negative results will be reported.

## Discussion

This trial evaluates the effectiveness of STATAMED compared to usual hospital care in terms of health and financial outcomes. Because this study is financed by the Innovation Fund of the German Federal Joint Committee, the evaluation will include a recommendation regarding the integration of STATAMED into the German health care system as part of routine care.

STATAMED will be implemented at six hospital sites. Being able to conduct a multisite trial represents a major advantage because the success of the intervention and evaluation will not depend on its implementation at a single location. Moreover, this multisite approach allows for the assessment of STATAMED across different settings, with three sites being located in rural and three in urban areas.

If STATAMED proves effective, it could represent a cost-effective and patient-friendly model of care, especially for elderly patients with chronic disease or acute infections (see eligibility criteria). The design of STATAMED, with its short-stay ward equipped with medical facilities to treat general medical conditions, appears suitable for widespread implementation. This model would be especially valuable for ensuring adequate health care provision in rural areas.

This study protocol reports the outcomes that will be measured using claims data from the participating statutory health insurers. In addition, other data will be collected as part of the overall project evaluation, such as from surveys among patients and referring entities, as well as interviews with STATAMED staff. These multiple perspectives and data sources will contribute to a comprehensive evaluation. The ability to link these data sources is an important strength of the study. For example, it will be possible to combine the claims data with survey results from participants. The ability to combine different data sets at the individual patient level offers opportunities to connect health outcomes and received treatments with patient-reported outcomes, thus enabling a deeper analysis and a better understanding of the trial results.

STATAMED comprises a unique combination of interventions, including a general medical short-stay hospital ward and a mandatory case review between the referring entity and the lead STATAMED physician, case managers, and mobile nurses. Analysing the effects of STATAMED thus represents an important contribution to existing research on health services. In addition to the claims data outlined in this protocol, STATAMED wards are required to document the services they provide, allowing for the measurement of the intensity of use. This will enable a more detailed assessment of the effectiveness of the interventions, moving beyond a simple binary measure of whether an intervention was applied or not.

One limitation of this study is that the project timeline does not allow for an investigation of the long-term health consequences of STATAMED for patients. Additionally, claims data will not be available for all patients who participate in the project; only data from those insured by one of the participating statutory health insurers will be available. This limitation could introduce selection bias, as the patient population of the participating health insurers may differ from the general population. However, access to demographic data will allow us to assess the generalisability of the results.

### Trial status

Recruitment started in April 2024. The current protocol is version 1, dated 17 July 2025. Recruitment is expected to continue until March 2026.

## Supplementary Information

Supplementary Material 1: Model consent form (participating health insurer).

Supplementary Material 2: Model consent form (non-participating health insurer).

## Data Availability

Due to data protection regulations in accordance with Book X of the German Social Code (SGB X), the data set cannot be made publicly available.

## Abbreviations

C-RCT: Cluster-randomised controlled trial
G-AEP: German Appropriate Evaluation Protocol
GEE: Generalised estimating equations
GLMM: Generalised linear mixed models
PCCL: Patient clinical complexity level
