# Supplementary material for: A cross-sectoral, short-stay hospital model in general medicine (STATAMED): study protocol for a cluster-randomised, stepped-wedge controlled trial
Source: Trials. 2025 Sep 16;26:340. doi: 10.1186/s13063-025-09072-6 (PMC12439374; doi:10.1186/s13063-025-09072-6)
Supplement: Supplementary file 1 — Supplementary Material 1: Model consent form (participating health insurer). [file 13063_2025_9072_MOESM1_ESM.pdf]

Health insurance fund or cost bearer		
Surname and first name of the insured person		Date of birth
Cost centre code	Insured person number	Status
Company number	Doctor no.	Date

Network number: 260

Appendix 3.1 – Declaration

for participation in the STATAMED contract pursuant to Section 140a of the German Social Code, Book V (SGB V)

I confirm that the participation requirements for the above-named insured person have been checked and are met.
In particular, I have verified that my patient is willing to actively cooperate and participate in the special care and will benefit from enrolment in terms of the agreed treatment goals.

Please enter today's date

M M M J J J Stamp of the registering authority Signature of the registering authority

1. Information on how to participate in this special provision

Your participation in this special care programme is voluntary. If you decide to participate, you are bound by your declaration of participation from the moment you sign it for the duration of the intervention phase. The intervention phase is currently scheduled to last for 24 months from 1 April 2024, but may be extended during the course of the project. However, you can cancel your participation with effect from the end of the quarter by notifying your health insurance fund. This does not affect your right to cancel your participation for good cause (e.g. change of residence, disrupted doctor-patient relationship, closure of the practice). Cancellation for good cause is possible without notice by writing to your health insurance fund and stating the reasons.

During your participation in this special care programme, your right to free choice of doctor for the contractual services only applies to the service providers participating in this contract. This restriction does not apply if you require a doctor or emergency service in a medical emergency. Your right to free choice of doctor for the treatment of other illnesses remains unaffected.

If, contrary to the above, you use another service provider not participating in this contract to provide the contractual services, or if you fail to comply with your obligations to cooperate as specified in detail in the insured person information, you may also be excluded from further participation in this care offer.

Your participation is also excluded if you are already participating in another contract for special care or a pilot project or innovation fund project of the health insurance fund for the same indication, which has the same objectives and provides the same benefits.

2. Declaration of participation

I hereby declare that

- I have been informed in detail about the contents, care objectives, reasons for termination and termination periods of the special care offer, I have been given the "Insured person's information on participation and data protection" from my health insurance fund regarding this care offer, and I agree with the contents thereof and the above information regarding my participation in this special care.
- I am particularly aware of what cooperation is required on my part and I am prepared to actively participate in the treatment.
- I am only entitled to benefits under this scheme if I am insured with a health insurance fund participating in this special scheme.
- I will inform my treating service provider of any change of health insurance fund.

Cancellation policy

You may revoke your declaration of participation within two weeks in writing, electronically or for the record at your health insurance company without giving reasons. To meet the deadline, it is sufficient to send the revocation declaration to the health insurance company in good time. The withdrawal period begins when your health insurance fund has informed you of your right of withdrawal in writing or electronically, but no earlier than when you submit your declaration of participation.

Yes, I would like to participate in the special care programme in accordance with the above information and confirm this with my signature.

3. Declaration of consent to data processing

I hereby declare that

- I consent to the processing of my medical and personal data collected in connection with the special care and I have received the "Insured Person Information on Participation and Data Protection" (attached to this form) and have taken note of the above information regarding my participation in this special care and scientific evaluation. I therefore release the doctors participating in STATAMED and any other participating members of the medical profession and their employees from their medical confidentiality obligations under Section 203 of the German Criminal Code (StGB) for the purpose of providing this special care. My consent is given voluntarily.
- I am aware that, regardless of the right of withdrawal under point 2, I may terminate my participation at the end of the quarter or without notice for good cause, and that the data collected and stored will be deleted upon my departure from the special pension scheme after expiry of the statutory periods.
- **I am aware that the data processing described in the "Insured Person Information on Participation and Data Protection" is a prerequisite for participation in the special care programme. I am also aware that consent to data protection is voluntary and that I can revoke it at any time in the future by contacting my health insurance provider (right of revocation pursuant to Art. 7 (3) GDPR), but that revocation will result in immediate termination of participation in the special care programme.**

Yes, I have received the "Insured Person Information on Participation and Data Protection" and have taken note of the above information regarding my participation in this special care programme. I consent to the processing of my treatment data as described therein in connection with my participation in the special care programme, release the doctors participating in STATAMED and any other participating members of the medical profession and their employees from their medical confidentiality obligations for the purpose of providing this special care, and confirm this with my signature.

Yes, I agree that the necessary information, in particular data from the patient file (surname, first name, date of birth, gender, address, contact details, insurance number, insurance code, insurance status, participation data, type of use, treatment day, contract data, prescription data and diagnosis), may be transferred to the insurance company for the purpose of billing and asserting the claim. date of birth, gender, address, contact details, insurance number, insurance fund code, insurance status, participation data, type of service used, date of treatment, contract data, prescription data and diagnoses according to ICD 10, service codes and their value, documented treatment data and - progress) may be exchanged between the billing centres and confirm this with my signature. The billing centres are participating doctors, hospitals, the AOK Rheinland/Hamburg health insurance fund and external billing service providers. External billing service providers (in accordance with the GDPR and BDSG) are of course also obliged to maintain confidentiality and to use the data for the specified purpose, as well as to comply with data protection and data security measures.

Yes, I agree that my data collected in the project from the patient file and routine data from my health insurance company may be transferred to the trust centre and scientific institutes in anonymised or pseudonymised form for evaluation and research purposes.

Please enter today's date Signature of the patient or legal representative

Wherever the masculine form is used in this Annex 3.1, this is for the sake of textual simplicity and includes all genders.

AOK
AOK Rheinland/Hamburg
Die Gesundheitskasse.

STATAMED

Insured person information on participation and data protection in the special STATAMED healthcare plan

<u>Contents, objectives and benefits of the pension agreement</u>

The innovative STATAMED care system breaks down the barriers between outpatient and inpatient treatment, allowing you to benefit from fewer changes of location, networked care and telemedical treatment. Targeted and planned short-term inpatient treatment and rapid discharge ensure that you can return to your familiar surroundings as quickly as possible. In addition, long-term treatment success is made possible by the cooperation of various professional groups and follow-up care in your home environment. This reduces the risk of you having to return to hospital. Our partners BürgerGesundheitsPark Bad Gandersheim, Klinikum Groß-Sand, Stadtteilklinik Hamburg, Krankenhaus Sulingen, Ubbo-Emmius-Klinik Norden and the St. Vincenz Health Centre stand for quality-assured medical care.

At STATAMED, experienced doctors, flying nurses and patient guides work in cooperation with your family doctor and specialists to provide you with care. The following diagrams illustrate the process.

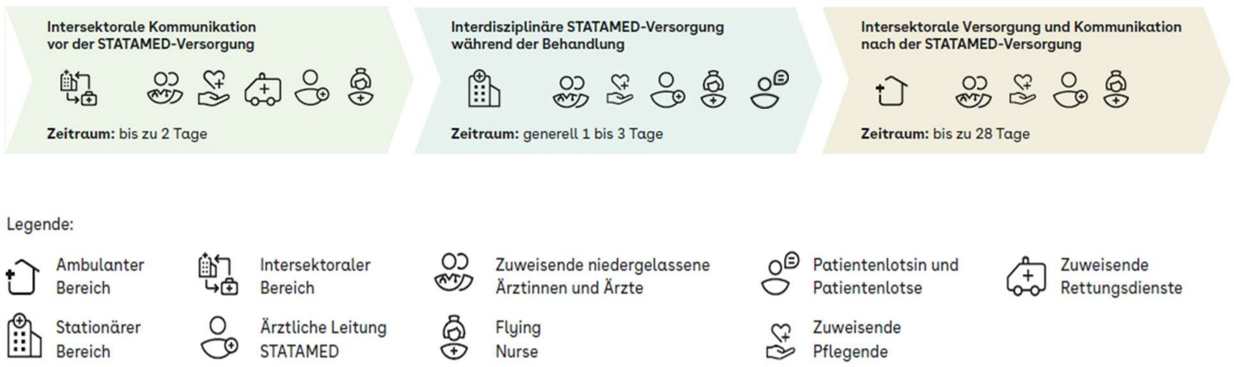

In the event of acute symptoms, care usually begins by contacting your family doctor. However, it is also possible for care to be provided by the emergency services, outpatient nursing services or nursing homes. The senior STATAMED doctors and the Flying Nurse are in close contact with the referring authorities. If necessary, you will be visited and examined in your home by a Flying Nurse, a mobile nurse equipped with telemedicine technology. The senior physician can be connected digitally and further treatment steps can be determined. As soon as you are admitted to the STATAMED clinic, you will be treated by the senior STATAMED physicians and welcomed by a patient guide. The patient navigator will be available to you as a contact person and will liaise with all regional professionals involved in or required for your treatment (general practitioners and specialists participating in the statutory health insurance system, nursing staff, MFAs, therapists, providers of therapeutic services, geriatric clinics, etc.).

day clinics, rehabilitation facilities) and cost bearers to ensure the success of treatment beyond discharge. This process is supported by the Flying Nurse, who can work at your location for up to four weeks after discharge to ensure the success of treatment and prevent readmissions. The extent to which the Flying Nurse is deployed is determined in consultation with your attending general practitioner or specialist.
This involves continuous communication between the various parties involved in the healthcare network.

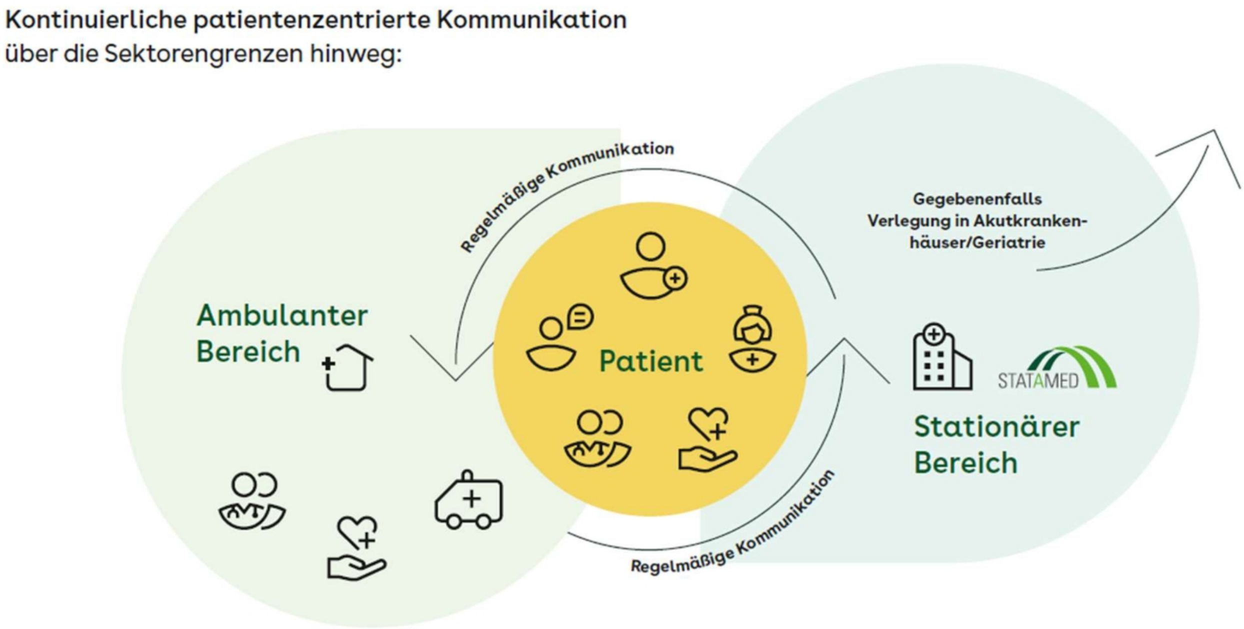

STATAMED is an innovation fund project for a new form of healthcare funded by the Joint Federal Committee (G-BA). As part of the STATAMED project, a scientific study must therefore be carried out. The aim is to conduct a comprehensive assessment (effectiveness, cost-effectiveness and acceptance) of the new needs-based and targeted form of healthcare. If the results are positive, STATAMED may be included as a regular and permanent service for insured persons in the German healthcare system. The Ethics Commission of the University of Hamburg and the Ethics Commission of the Hannover Medical School have approved the scientific evaluation.

Scientific evaluation and scientific project support are provided by the following institutions:

External evaluation:

- Hamburg Center for Health Economics (HCHE), University of Hamburg: Summative evaluation, formative evaluation, health economic evaluation and SEIA.
Address: Hamburg Center for Health Economics (HCHE) Esplanade 36 20354 Hamburg
Email: evaluation-statamed.hche@uni-hamburg.de
- Hannover Medical School, MHH – Institute for General Medicine and Palliative Care: Formative evaluation and qualitative process evaluation Hannover Medical School, MHH – Address: Institute for General Medicine and Palliative Care, Carl-Neuberg-Str. 1, 30625 Hannover, Germany Telephone: +49 511 532-4997 Email: statamed@mh-hannover.de

Scientific project support

- University Medical Centre Hamburg-Eppendorf: Quality assurance and operationalisation of the continuous improvement process, Address: University Medical Centre Hamburg-Eppendorf, Centre for Psychosocial Medicine, Institute and Polyclinic for General Medicine, Martinistraße 52, 20246 Hamburg
- Institute for Health Care Business GmbH (hcb): Economic evaluation and framework conditions for successful transfer to standard care Address: Institute for Health Care Business GmbH, Friedrich-Ebert-Straße 55, 45127 Essen, email: info@hcb-institute.de

Period of participation

Your participation begins on the day you sign the declaration of participation. **From this point on, you are bound by your declaration of participation for the duration of the intervention phase. The intervention phase is currently scheduled to last for 24 months from 1 April 2024, but may be extended during the course of the project.** Participation can be terminated with effect from the end of the quarter. Participation also ends when you switch to a health insurance fund that is not party to the contract or to a service provider that is not participating in the contract. In this case, it is important to inform your current health insurance fund and, if applicable, your patient navigator about your change of health insurance fund. Participation also ends when your health insurance provider or the treating physician withdraws from this contract. Furthermore, your participation also ends if you revoke your participation or your consent to data processing or if you fail to fulfil your obligations to cooperate.
Any revocation or cancellation must be sent to your health insurance provider.
Those insured by AOK Rheinland/Hamburg should address it to AOK Rheinland/Hamburg – Die Gesundheitskasse Bereich Gesundheitsmanagement, Abteilung Versorgungsinnovation, Kasernenstr. 61, 40213 Düsseldorf
If you are insured with AOK Lower Saxony, please send your cancellation to AOK – Die Gesundheitskasse für Niedersachsen, Unternehmensbereich Strukturierte Versorgung, Hans-Böckler-Allee 13, 30173 Hannover.

Obligation to cooperate and consequences of failure to cooperate

Once you have confirmed your participation in the special care programme, you are committed to participating for the duration of the intervention phase. The intervention phase is currently scheduled to last for a period of 24 months from 1 April 2024, but may be extended during the course of the project. In each acute case, a reassessment will be carried out to determine whether treatment within the framework of STATAMED is possible. If home visits by the Flying Nurse are agreed with you after your discharge, you are obliged to keep to the agreed appointments or to inform them in good time if you are unable to attend. Another component of participation in the project is the completion of questionnaires. In addition, please inform your current health insurance provider and your patient navigator if you change health insurance providers. Repeated violations of these obligations to cooperate may result in exclusion from the contract.

Billing for treatment services

There are no costs for participating in STATAMED. For billing purposes, your participation data will be forwarded to AOK Rheinland/Hamburg, which will claim the subsidies. For this reason, billing data for treatment services will also be forwarded to AOK Rheinland/Hamburg by the Association of Statutory Health Insurance Physicians in North Rhine-Westphalia and the clinics participating in STATAMED.

Data

What data do we require from you?

Cooperation between your health insurance provider and other contractual partners, such as doctors and hospitals, enables individual treatment processes to be coordinated in order to guarantee smooth procedures and high-quality medical care. The data processing described below is necessary for this purpose and is a prerequisite for your participation. Only personal data that is absolutely necessary for the lawful processing of the procedure will be collected. The statutory data protection regulations – in particular the provisions of the General Data Protection Regulation (EU GDPR) and the Social Security Code (SGB) – are observed at every stage of processing. The protection of your data is always guaranteed! Only employees who have been specially selected and trained for the provision of special care have access to your data.

Data held by our contractual partners

For the purpose of fulfilling the treatment contract and your participation in STATAMED, personal health data will be collected and processed by the service providers. This includes data about the type and results of your treatment (e.g. severity of the illness, duration of treatment, type of treatment, hospitalisation, medication) and information about your state of health contained in your patient file held by your treating service providers at the STATAMED facility will only be accessed by the contractual partners and, in the event of complications, by the medical service (except for billing purposes in accordance with data protection regulations). The documentation may be stored in an electronic patient file maintained, managed and supervised by the STATAMED facility. Your health insurance company will not receive any findings data. The respective STATAMED facility is responsible for data processing. If you have any questions about this data processing, please contact your treating service provider.

It is intended that the data collected will be transmitted in pseudonymised form, i.e. without names being mentioned, but in coded form (e.g. random combinations of numbers/letters), so that no conclusions can be drawn about your identity, for the purposes of scientific monitoring/evaluation to improve the care situation of persons with statutory health insurance by the contractual partners to the appointed trust centre. The trusted third party is commissioned to transmit the collected data to the scientific evaluation institutions (see above) for evaluation and assessment of the new STATAMED form of care. The use of pseudonymised data is essential for the scientific monitoring/evaluation of the components of STATAMED care. It will be made available exclusively for the purpose of monitoring the success of the STATAMED care programme and for scientific purposes.

The North Rhine Association of Statutory Health Insurance Physicians is also a contractual partner in the project and involves doctors participating in statutory health insurance. As part of the project, your participation, treatment services and billing will be recorded and pseudonymised for scientific monitoring/evaluation. The North Rhine Association of Statutory Health Insurance Physicians is responsible for processing this data.

Data held by your health insurance fund

Your health insurance provider will only receive information about your participation and the services you have received as part of the special care programme. The data will be processed for the fulfilment of legal obligations in accordance with Section 284 (1) No. 13 of the German Social Code, Book V (SGB V) in conjunction with Section 140a SGB V and used for other legal purposes, such as billing verification. Your health insurance provider is responsible for this data processing. Recipients of your data may be third parties or commissioned service providers within the scope of their legal obligations and powers of disclosure. The data will be stored for the purpose of performing the tasks and for the duration of the statutory retention periods (e.g. Section 110a SGB IV, Section 304 SGB V, Section 107 SGB XI) and then deleted.

You have the following rights with regard to data protection:

- The right to information about processed data (Art. 15 GDPR in conjunction with Section 83 SGB X)
- The right to rectify inaccurate data (Art. 16 GDPR in conjunction with Section 84 SGB X)
- The right to erasure of your data (Art. 17 GDPR in conjunction with § 84 SGB X)
- The right to restrict the processing of your data (Art. 18 GDPR in conjunction with § 84 SGB X)
- The right to data portability (Art. 20 GDPR in conjunction with Section 84 SGB X)
- The right to object (Art. 21 GDPR in conjunction with Section 84 SGB X)
- The right to withdraw your consent at any time with future effect. This does not affect the lawfulness of data processing carried out on the basis of your consent until withdrawal.

Contact person for questions about the project, data processing or data protection:

If you have any questions about the project, please contact your healthcare provider or health insurance company at any time. If you are insured with AOK Rheinland/Hamburg, please contact them at statamed@rh.aok.de. If you are insured with AOK Niedersachsen, please contact your health insurance company atStatAMed@nds.aok.de .

If you have any doubts about the legality of the processing of your personal data, you have the right to lodge a complaint with the State Commissioner for Data Protection and Freedom of Information of North Rhine-Westphalia, Kavalleriestr. 2-4, 40213 Düsseldorf,poststelle@ldi.nrw.de , the data protection supervisory authority of AOK Rheinland/Hamburg. If you have any questions, please contact your health insurance fund or its data protection officer, for insured persons of AOK Rheinland/Hamburg Kasernenstr. 61, 40213 Düsseldorf,datenschutz@rh.aok.de .

Participants in Lower Saxony can contact the State Commissioner for Data Protection in Lower Saxony, Prinzenstraße 5, 30519 Hanover. If you have any questions, please contact the data protection officer of AOK Lower Saxony, Hildesheimer Straße 273, 30519 Hanover,Datenschutz@nds.aok.de .

If you have any questions about the scientific evaluation, please contact the above-mentioned

institutes. Scientific evaluation

For the scientific evaluation of the care programme, your health insurance fund will transfer your already stored data (master data, type of insurance, periods of incapacity for work and costs with diagnoses, outpatient billing and service data, hospital stays and costs with diagnoses, preventive and rehabilitation measures with diagnoses and costs, duration and costs of home nursing care, type and costs of prescribed medicines, remedies and aids, travel costs, nursing services and costs, and any data stored as part of participation in the Chronic Disease Management Programme (DMP) to a designated trusted third party in pseudonymised form. For the purpose of evaluating the new form of care, it links the collected and stored data (data linkage) and makes a complete data set available to scientific institutions for evaluation. The basis for the linkage is an insured person pseudonym and an institution pseudonym. The data is transmitted exclusively in encrypted form using state-of-the-art technology. A project-specific data protection concept is in place that complies with the federal and state-specific provisions of German data protection law and the EU General Data Protection Regulation. The data will be treated confidentially at all times and will be made available exclusively for quality control, evaluation of the STATAMED care programme and scientific purposes. The evaluating institutions will evaluate the data in anonymised form. Scientific publications of results will be made exclusively in anonymised form, i.e. in a form that does not allow any conclusions to be drawn about your person.

In order to evaluate the new STATAMED care model overall, additional surveys will be conducted to assess your satisfaction and the success of your treatment. You will be asked to give your consent to these surveys at a later date in a separate consent form.

All data is stored and archived in a secure system for a period of ten years in accordance with scientific standards. Your data will then be deleted or anonymised, unless legal reasons require longer storage. Anonymised means that the allocation code is deleted and the data cannot be traced back to you in any way.
