# Supplementary material for: A cross-sectoral, short-stay hospital model in general medicine (STATAMED): study protocol for a cluster-randomised, stepped-wedge controlled trial
Source: Trials. 2025 Sep 16;26:340. doi: 10.1186/s13063-025-09072-6 (PMC12439374; doi:10.1186/s13063-025-09072-6)
Supplement: Supplementary file 2 — Supplementary Material 2: Model consent form (non-participating health insurer). [file 13063_2025_9072_MOESM2_ESM.pdf]

Health insurance fund or cost bearer		
Surname and first name of the insured person		Date of birth
Cost centre code	Insured person number	Status
Company number	Doctor no.	Date

Network number: 260

Appendix 3.2 – Declaration

for participation in the treatment contract pursuant to Section 630 a of the German Civil Code (BGB) "STATAMED"

I confirm that the participation requirements for the above-named insured person have been checked and are met.
In particular, I have verified that my patient is willing to actively cooperate and participate in STATAMED and can benefit from participation with regard to the agreed therapy goals.

Please enter today's date

M M Y J J J | Stamp of the registering authority | Signature of the registering authority

1. Information on participating in STATAMED

Your participation in STATAMED is voluntary. If you decide to participate, you are bound by your declaration of participation from the moment you sign it for the duration of the intervention phase. The intervention phase is currently scheduled to last for 24 months from 1 April 2024, but may be extended during the course of the project. However, you may terminate your participation with effect from the end of the quarter at any of the clinics participating in STATAMED. This does not affect your right to terminate your participation for good cause (e.g. change of residence, disrupted doctor-patient relationship, closure of practice). Termination for good cause is possible without notice in writing, stating the reasons, to the clinic participating in STATAMED.

During your participation in STATAMED, your right to free choice of doctor for the use of the contractual services only applies to the service providers participating in this contract. This restriction does not apply if you require a doctor or emergency service in a medical emergency. Your right to free choice of doctor for the treatment of other illnesses remains unaffected.

If, contrary to the above, you use another service provider who is not participating in this contract to provide the contractual services, or if you fail to comply with your obligations to cooperate as specified in the insured person information, you may also be excluded from further participation in this care programme.

2. Declaration of participation

I hereby declare that

- I have been informed in detail about the contents, treatment goals, reasons for termination and termination periods of STATAMED, I have been given the "Insured Person Information on Participation and Data Protection" for the STATAMED project, and I agree to the contents thereof and to the above information regarding my participation in STATAMED. I hereby declare my consent to participate in the treatment contract in accordance with Section 630a of the German Civil Code (BGB) for the STATAMED project. This treatment contract is part of the STATAMED innovation fund project.
- I am particularly aware of what cooperation is required on my part and I am prepared to actively participate in the treatment.

Right of withdrawal

You may withdraw your declaration of participation within two weeks in writing, electronically or for the record at the clinics participating in STATAMED without giving reasons. To meet the deadline, it is sufficient to send the declaration of withdrawal to the STATAMED locations in good time. The withdrawal period begins when the hospital has informed you of your right of withdrawal in writing or electronically, but no earlier than when you submit your declaration of participation.

Yes, I would like to participate in STATAMED in accordance with the above information and confirm this with my signature.

3. Declaration of consent to data processing

I hereby declare that

- I consent to the processing of my medical and personal data collected in the special care system and I have received the "Insured Person Information on Participation and Data Protection" (attached to this form) and have taken note of the above information on my participation in STATAMED and on the scientific evaluation. I therefore release the doctors who have joined STATAMED, as well as any other participating members of other healthcare professions and their employees, from their medical confidentiality obligations under Section 203 of the German Criminal Code (StGB) for the purposes of STATAMED. My consent is given voluntarily.
- I am aware that, regardless of the right of withdrawal in accordance with point 2, I can terminate my participation at the end of the quarter or without notice for good cause and that the data collected and stored will be deleted upon my departure from STATAMED after the expiry of the statutory periods.
- I am aware that the data processing described in the "Insured Person Information on Participation and Data Protection" is a prerequisite for participation in STATAMED. I am also aware that consent to data protection is voluntary and that I can revoke it at any time by sending an email to AOK Rheinland/Hamburg atstatamed@rh.aok.de , but that revocation will result in immediate termination of participation in STATAMED.

Yes, I have received the "Insured Person Information on Participation and Data Protection" and have taken note of the above information regarding my participation in STATAMED. I consent to the processing of my treatment data as described therein within the scope of my participation in the special care programme, release the doctors joining STATAMED and any other participating members of other healthcare professions and their employees from their medical confidentiality obligations for the purposes of STATAMED, and confirm this with my signature.

Yes, I agree that the necessary information, in particular data from the patient file (surname, first name, date of birth, gender, address, contact details, insurance number, insurance code, insurance status, participation data, type of use, treatment date, contract data, prescription data and diagnosis), may be transferred to the insurance company for the purpose of billing and asserting the claim. date of birth, gender, address, contact details, insurance number, insurance fund code, insurance status, participation data, type of service used, date of treatment, contract details, prescription details and diagnoses according to ICD 10, service codes and their value, documented treatment data and treatment history) may be exchanged between the billing centres for the purpose of billing and asserting the claim, and I confirm this with my signature. The billing centres are participating doctors, hospitals, the AOK Rheinland/Hamburg health insurance fund and external billing service providers. External billing service providers (in accordance with the GDPR and BDSG) are of course also obliged to maintain confidentiality and to use the data for the specified purpose, as well as to comply with data protection and data security measures.

Yes, I agree that my data collected in the project from the patient file may be transferred to a trusted party and scientific institutes for evaluation purposes.

Please enter today's date | Signature of the patient or legal representative

Wherever the masculine form is used in this Appendix 3.2, this is for the sake of textual simplicity and includes all genders.

Insured person information on participation and data protection in the special form of care STATAMED

Contents, objectives and services of the STATAMED project

The innovative STATAMED care system breaks down the barriers between outpatient and inpatient treatment, allowing you to benefit from fewer changes of location, networked care and telemedical treatment. Targeted and planned short-term inpatient treatment and rapid discharge ensure that you can return to your familiar surroundings as quickly as possible. In addition, long-term treatment success is made possible by the cooperation of various professional groups and follow-up care in your home environment. This reduces your risk of having to return to hospital. Our partners BürgerGesundheitsPark Bad Gandersheim, Klinikum Groß-Sand, Stadtteilklinik Hamburg, Krankenhaus Sulingen, Ubbo-Emmius-Klinik Norden and Gesundheitszentrum Essen Stoppenberg stand for quality-assured medical care.

At STATAMED, experienced doctors, flying nurses and patient guides take care of you in cooperation with your family doctor and specialists. The following diagrams illustrate the process.

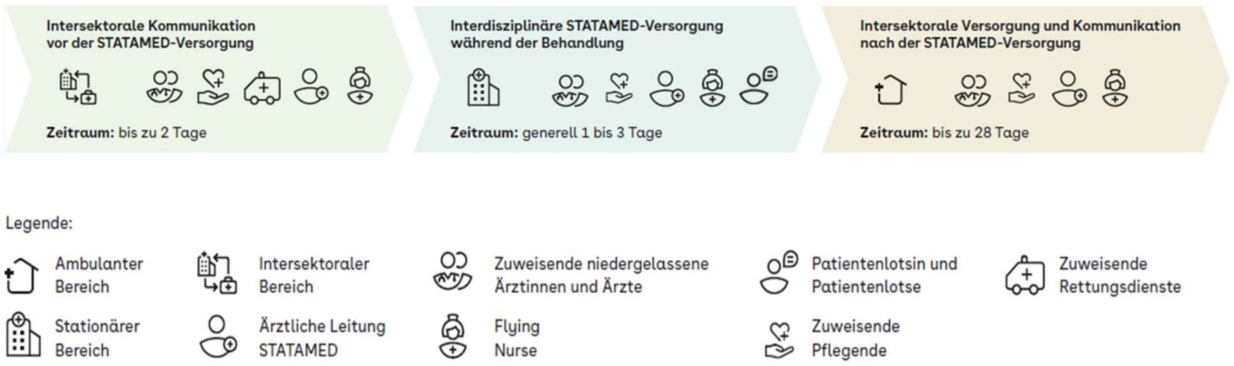

In the event of acute symptoms, care usually begins by contacting your family doctor. However, it is also possible for care to be provided by the emergency services, outpatient nursing services or nursing homes. The senior STATAMED doctors and the Flying Nurse are in close contact with the referring authorities. If necessary, you will be visited and examined in your home by a Flying Nurse, a mobile nurse equipped with telemedicine technology. The senior physician can be connected digitally and the next steps in your treatment will be determined. As soon as you are admitted to the STATAMED clinic, you will be treated by the senior STATAMED physicians and welcomed by a patient guide. The patient navigator will be available to you as a contact person and will liaise with all regional professionals involved in or required for your treatment (registered general practitioners and specialists, nursing staff, MFAs, therapists, providers of therapeutic remedies, geriatric clinics or day clinics, rehabilitation facilities).

and cost bearers to ensure the success of treatment beyond discharge. This process is supported by the Flying Nurse, who can work at your home for up to four weeks after discharge to ensure the success of treatment and prevent readmissions. The extent to which the Flying Nurse is deployed is determined in consultation with your attending general practitioner or specialist.
This involves continuous communication between the various parties involved in the healthcare network.

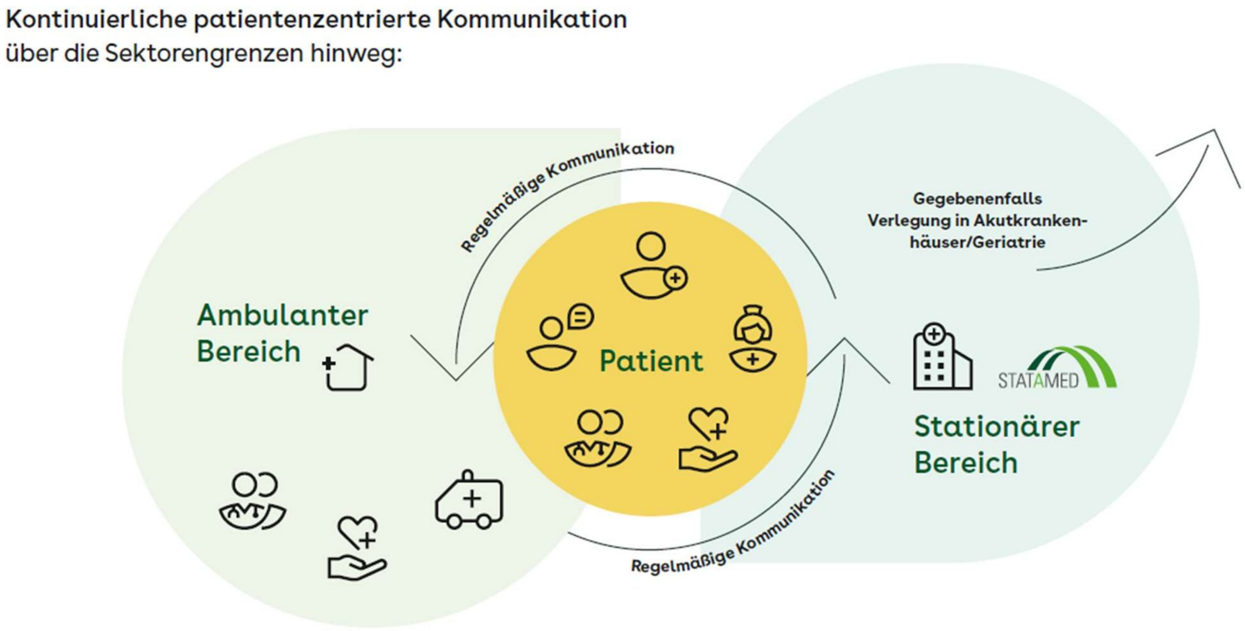

STATAMED is an innovation fund project for a new form of healthcare funded by the Joint Federal Committee (G-BA). As part of the STATAMED project, a scientific study must therefore be conducted. The aim is to comprehensively evaluate (effectiveness, cost-effectiveness and acceptance) this new needs-based and targeted form of healthcare. If the results are positive, STATAMED may be included as a regular and permanent service for insured persons in the German healthcare system. The Ethics Committee of the University of Hamburg and the Ethics Committee of the Hannover Medical School have approved the scientific evaluation.

The scientific evaluation and scientific project support will be carried out by the following institutions:

External evaluation:

- Hamburg Centre for Health Economics (HCHE), University of Hamburg: Summative evaluation, formative evaluation, health economic evaluation and SEIA.
Address: Hamburg Centre for Health Economics (HCHE) Esplanade 36 20354 Hamburg
Email: evaluation-statamed.hche@uni-hamburg.de
- Hannover Medical School, MHH – Institute for General Medicine and Palliative Medicine: Formative evaluation and qualitative process evaluation Hannover Medical School, MHH – Address: Institute for General Medicine and Palliative Medicine, Carl-Neuberg-Str. 1, 30625 Hannover Telephone: +49 511 532-4997 Email: statamed@mh-hannover.de

Scientific project support

- University Medical Centre Hamburg-Eppendorf: Quality assurance and operationalisation of the continuous improvement process, Address: University Medical Centre Hamburg-Eppendorf, Centre for Psychosocial Medicine, Institute and Polyclinic for General Medicine, Martinistraße 52, 20246 Hamburg
- Institute for Health Care Business GmbH (hcb): Economic evaluation and framework conditions for successful transfer to standard care Address: Institute for Health Care Business GmbH, Friedrich-Ebert-Straße 55, 45127 Essen, email: info@hcb-institute.de

Period of participation

Your participation begins on the day you sign the declaration of participation. From this point on, you are bound by your declaration of participation for the duration of the intervention phase. The intervention phase is currently scheduled to last for 24 months from 1 April 2024, but may be extended during the course of the project. Participation can be terminated with effect from the end of the quarter. Participation also ends with the end of membership in statutory health insurance or with a change to a service provider not participating in the contract. In addition, your participation will also end if you revoke your participation or your consent to data processing or if you fail to fulfil your obligations to cooperate.

Obligation to cooperate and consequences of failure to cooperate

Once you have confirmed your participation in the special care programme, you are committed to participating for the duration of the intervention phase. The intervention phase is currently scheduled to last for a period of 24 months from 1 April 2024, but may be extended during the course of the project. In each acute case, a reassessment will be carried out to determine whether treatment within the framework of STATAMED is possible. If home visits by the Flying Nurse are agreed with you after your discharge, you are obliged to keep to the agreed appointments or to inform them in good time if you are unable to attend. Another component of participation in the project is the completion of questionnaires. In addition, please inform your patient navigator if your membership in the statutory health insurance scheme ends. Repeated violations of these obligations to cooperate may result in exclusion from STATAMED.

Billing for treatment services

There are no costs for participating in STATAMED. For billing purposes, your participation data will be forwarded to AOK Rheinland/Hamburg, which will claim the subsidies. For this reason, billing data for treatment services will also be forwarded to AOK Rheinland/Hamburg by the clinics participating in STATAMED. The billing of care services provided by outpatient general practitioners and specialists is carried out by a billing service provider selected for STATAMED care and contractually bound to AOK Rheinland/Hamburg.

Data protection

What data do you need from me?

The cooperation between AOK Rheinland/Hamburg as project manager and other contractual partners, such as doctors and hospitals, enables the individual treatment processes to be coordinated in order to guarantee smooth procedures and high-quality medical care. The data processing described below is necessary for this purpose and is a prerequisite for your participation. Only personal data that is absolutely necessary for the lawful processing of the process will be collected. The statutory data protection regulations – in particular the provisions of the General Data Protection Regulation (EU GDPR) and the Social Security Code (SGB) – are observed at every stage of processing. The protection of your data is always guaranteed! Only employees who have been specially selected and trained for special care have access to your data.

Data held by our contractual partners

For the purpose of fulfilling the treatment contract and your participation in STATAMED, personal health data will be collected and processed by the service providers. This includes data about the type and results of your treatment (e.g. severity of the illness, duration of treatment, type of treatment, hospital days, medication) and the information about your state of health contained in your patient file held by your treating service providers at the STATAMED facility will only be accessed by the contractual partners and, in the event of possible complications, by the medical service (except for billing purposes in accordance with data protection regulations). The documentation may be stored in an electronic patient file maintained, managed and supervised by the STATAMED facility. Your health insurance company will not receive any findings data. The respective STATAMED facility is responsible for data processing. If you have any questions about this data processing, please contact your treating service provider.

It is intended that the data collected will be transmitted in pseudonymised form, i.e. without names being mentioned, but in coded form (e.g. random combinations of numbers/letters), so that no conclusions can be drawn about your identity, for the purposes of scientific monitoring/evaluation to improve the care situation of persons with statutory health insurance by the contractual partners to the appointed trust centre. The trusted third party is commissioned to transmit the collected data to the scientific evaluation institutions (see above) for evaluation and assessment of the new STATAMED form of care. The use of pseudonymised data is essential for the scientific monitoring/evaluation of the components of STATAMED care. It will be made available exclusively for the purpose of monitoring the success of the STATAMED care programme and for scientific purposes.

As part of the project, your participation, treatment services and invoices will be recorded and pseudonymised for scientific monitoring/evaluation.

In order to pay for the services provided by the participating general practitioners and specialists, an external billing service provider may be used, which is contractually bound to AOK Rheinland/Hamburg. For this purpose, the billing service provider receives personal service data collected by the STATAMED hospitals. In addition, the participating general practitioners and specialists are informed about their participation in the project.

Data at AOK Rheinland/Hamburg

As the consortium leader for the project, AOK Rheinland/Hamburg only receives information about your participation and the services you have received within the scope of the project. The data will be processed and used to fulfil reporting obligations to the funding body. AOK Rheinland/Hamburg is responsible for this data processing. The data will be stored for the purpose of performing the tasks and for the duration of the statutory retention periods (e.g . Section 110a of the German Social Code, Book IV (SGB IV), Section 304 of the German Social Code, Book V (SGB V), Section 107 of the German Social Code, Book XI (SGB XI)) and then deleted.

You have the following rights with regard to data protection:

- The right to information about processed data (Art. 15 GDPR in conjunction with Section 83 SGB X)
- The right to rectify inaccurate data (Art. 16 GDPR in conjunction with Section 84 SGB X)
- The right to erasure of your data (Art. 17 GDPR in conjunction with Section 84 SGB X)
- The right to restrict the processing of your data (Art. 18 GDPR in conjunction with Section 84 SGB X)
- The right to data portability (Art. 20 GDPR in conjunction with Section 84 SGB X)
- The right to object (Art. 21 GDPR in conjunction with Section 84 SGB X)
- The right to withdraw your consent at any time with future effect. This does not affect the lawfulness of data processing based on your consent until withdrawal.

Contact person for questions about the project, data processing or data protection:

If you have any questions about the project, you can contact your service provider or AOK Rheinland/Hamburg at any time at the email addressstatamed@rh.aok.de .

If you have any questions about data protection or your rights as a data subject, please contact the data protection officer at AOK Rheinland/Hamburg Kasernenstr. 61, 40213 Düsseldorf,datenschutz@rh.aok.de . If you have any doubts about the lawfulness of the processing of your personal data, you have the right to lodge a complaint with the State Commissioner for Data Protection and Freedom of Information of North Rhine-Westphalia, Kavalleriestr. 2-4, 40213 Düsseldorf,poststelle@ldi.nrw.de , the data protection supervisory authority of AOK Rheinland/Hamburg.

If you have any questions about the scientific evaluation, please contact the above-mentioned institutes.

Scientific evaluation

For the purpose of evaluating the new form of care, the trust centre links the collected and stored data (data linkage) and makes a complete data set available to scientific institutions for evaluation. The basis for the linkage is an insured person pseudonym and an institution pseudonym. The data is transmitted exclusively in encrypted form using state-of-the-art technology. A project-specific data protection concept is in place that complies with the federal and state-specific provisions of German data protection law and the EU General Data Protection Regulation. The data will be treated confidentially at all times and will be used exclusively for quality control, evaluation of the STATAMED care programme and

scientific purposes. The evaluating institutions will evaluate the data anonymously. Scientific publications of results will be made exclusively in anonymised form, i.e. in a form that does not allow any conclusions to be drawn about your person.

In order to evaluate the new STATAMED care model as a whole, additional surveys will be conducted to assess your satisfaction and the success of your treatment. You will be asked to give your consent to these surveys at a later date in a separate consent form.

All data will be stored and archived in a secure system for a period of ten years in accordance with scientific standards. Your data will then be deleted or anonymised, unless longer storage is required by law. Anonymised means that the allocation code will be deleted and the data cannot be traced back to you in any way.
